# A systematic review and meta-analysis of 235delC mutation of *GJB2* gene

**DOI:** 10.1186/1479-5876-10-136

**Published:** 2012-07-02

**Authors:** Jun Yao, Yajie Lu, Qinjun Wei, Xin Cao, Guangqian Xing

**Affiliations:** 1 Department of Biotechnology, School of Basic Medical Science, Nanjing Medical University, Nanjing, 210029, People’s Republic of China; 2 The State Key Laboratory of Reproductive Medicine, Department of Biotechnology, Nanjing Medical University, Hanzhong Road No.140, Nanjing, 210029, People’s Republic of China; 3 Department of Otorhinolaryngology, The First Affiliated Hospital of Nanjing Medical University, Guangzhou Road No.300, Nanjing, 210029, People’s Republic of China

**Keywords:** *GJB2*, 235delC, Non-syndromic hearing loss, Meta-analysis

## Abstract

**Background:**

The 235delC mutation of *GJB2* gene is considered as a risk factor for the non-syndromic hearing loss (NSHL), and a significant difference in the frequency and distribution of the 235delC mutation has been described world widely.

**Methods:**

A systematic review was performed by means of a meta-analysis to evaluate the influence of the 235delC mutation on the risk of NSHL. A literature search in electronic databases using keywords “235delC”, “*GJB2*” associated with “carrier frequency” was conducted to include all papers from January 1999 to June 2011. A total of 36 papers were included and there contained 13217 cases and 6521 controls derived from Oceania, American, Europe and Asian.

**Results:**

A remarkable heterogeneity between these studies was observed. The combined results of meta-analysis showed that the 235delC mutant increased the risk of NSHL (*OR* = 7.9, 95%*CI* 4.77 ~ 13.11, *P* <0.00001). Meanwhile, heterogeneity of genetic effect was also observed due to the ethnic specificity and regional disparity. Therefore, the stratified meta-analysis was subsequently conducted and the results indicated that the 235delC mutation was significantly correlated with the risk of NHSL in the East Asian and South-east Asian populations (*OR* = 12.05, 95%*CI* 8.33~17.44, *P* <0.00001), but not significantly in the Oceania and European populations (*OR* = 10.36, 95%*CI*: 4.68~22.96, *Z* = 1.68, *P* >0.05).

**Conclusions:**

The 235delC mutation of *GJB2* gene increased the risk of NHSL in the East Asian and South-east Asian populations, but non-significantly associated with the NSHL susceptibility in Oceania and European populations, suggesting a significant ethnic specificity of this NSHL-associated mutation.

## Introduction

Hearing impairment is the most common sensory disorder, present in 1 of every 1000 newborns, about half of which could be attributed to genetic factors [1]. There are two monogenic forms of hearing loss including syndromic (characterized by hearing loss in combination with other abnormalities) and non-syndromic (with only hearing loss) deafness [1]. Up to now, more than 70 loci for non-syndromic hearing loss (NSHL) had been identified (http://hereditaryhearingloss.org/). Among them, DFNB1 was considered to be the main cause of prelingual deafness. As one of the DFNB1 locus-linked genes, *GJB2* was the first to be indentified which encoded the gap junction protein connexin 26 (Cx26). To date, more than a hundred different mutations of this gene has been implicated in NSHL [2]. The most common mutations of *GJB*2 gene comprised 35delG, 167delT and 235delC with the varied incidence among populations [2].

The 235delC mutation of *GJB2* is the most frequently known mutation in some East Asian groups, with a carrier frequency of approximately 1% [3]. Recently, a substantial amount of research had been devoted to elucidate the influence of this mutation on the risk of NSHL, but conflicting results were obtained. Some studies reported that the 235delC mutation had only limited effect on the risk of NSHL [4,5], while others suggested that the 235delC mutation increased the risk of NSHL or was concerned with the specificity pathological type of NSHL [6,7]. In those reported studies, the genetic background of the ethnic specificity and the regional disparity were not completely considered, or small effects in human genetic association were difficult to be detected due to small sample size of case-control studies. To clarify the variable results, a further research on the association between ethnic specificity of the 235delC mutation and the NSHL susceptibility was essentially needed. In this study, a combined meta-analysis was performed by pooling data from all relevant case-control studies published in recent ten years. The association of the 235delC mutation with hearing loss and the heterogeneity of genetic effect were evaluated, based on which the stratified meta-analysis was subsequently conducted to assess the ethnic specificity of the 235delC mutation associated with the NSHL susceptibility.

## Methods

### Literature search

The electronic databases including PubMed, InterScience, British Library Direct, Embase, Sciencedirect, Chinese Biomedical Literature, Chinese WanFang database and China National Knowledge Infrastructure were searched from January 1999 to June 2011, fulfilling all case-control studies holding information on the epidemiology study in molecular genetics of hearing loss. The following terms were used in search strategies: *GJB2*, Connexin 26 (Cx26), mutation, variant, NSHL or NSHI (non-syndromic hearing impairment). All related citations were retrieved to find other relevant articles that were not initially identified. The entire literature search was performed by two independent researchers.

### Study selection

The reference lists of all traced articles were examined manually and screened for eligibility using the following criteria: (1) the association of the 235delC mutation of *GJB2* with NSHL was examined based on case-control design; (2) the selected papers contained complete information and represented Odds Ratio (*OR*) and 95% Confidence Interval (95%*CI*) directly or indirectly; (3) for the same studied populations, only the papers newly published or providing the complete data were adopted. All collected papers were scored and categorized according to the Newcastle-Ottawa Scale (NOS) for assessing the quality of case-control studies [8]. Studies with the low score (below 6) or the genetic type of control group inconsistent with the principle of Hardy-Weinberg Equilibrium (HWE) were categorized to Grade B, and the others were categorized to Grade A [9].

### Data extraction

**Figure 1:**
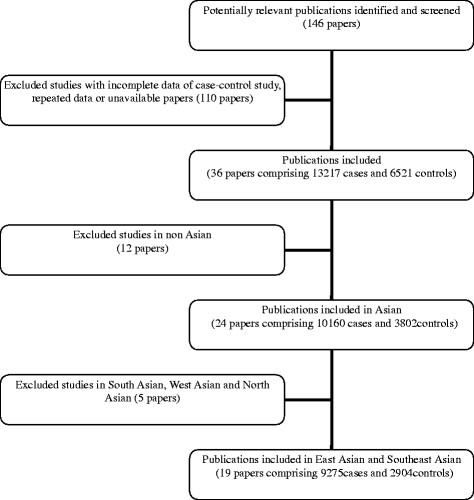
Flow chart of review process.

A total of 146 papers were obtained through the search of electronic databases. Among them, 36 papers associated with the 235delC mutation of *GJB2* gene and the NSHL susceptibility were selected for the following data analysis. The flow chart of review process was shown as Figure 1.

### Data analysis

The mean value of carrier frequency of the 235delC mutation in the individual studies was calculated. The included populations were divided into two groups (Asian and non-Asian groups) according to the distribution of carriers. The relationship between the 235delC mutation of *GJB2* and hearing loss was evaluated for each group or continent. The statistical analysis was conducted using Review Manager 5.1 (RevMan) supplied by the Cochrane Collaboration (Oxford, UK). Heterogeneity among studies was assessed through Cochrane χ^2^ test and *I*^2^ statistic (*P* < 0.05 indicating the existence of between-study heterogeneity). Based on the test of heterogeneity, the combined meta-analysis and stratified meta-analysis were conducted respectively. The pooled *OR* was calculated using fixed or random effect model, along with the 95%*CI* to measure the strength of the genetic association. The publication bias was analyzed by STATA 11.0 and Begg’s test, and failsafe number (*N*_f*s*_) was calculated (*N*_f*s*_ > 10, *P* > 0.05 means that the obtained conclusion was confident). The statistical analysis was performed using Software SPSS (Version 13, Chicago, USA). An online tool (http://ihg.gsf.de/cgi-bin/hw/hwa1.pl/) was applied to test whether the genetic type of control group was consistent with Hardy-Weinberg equilibrium (HWE) for each study.

## Results

### The 235delC carrier frequency of GJB2 allele

Among 36 included papers, there were 24 papers containing the information on the 235delC carrier frequencies of *GJB2* allele, as shown in Table 1.

**Table 1:** **235del mutation frequencies of of*GJB2*allele in the individual studies**

**No**	**Relevant Studies**	**TOTAL**	**HETRO**	**HOMO**	**TOTAL%**
1	Ohtsuka A, *et al*. 2003 [10]	1227	92	16	5.05%
2	Batissoco AC, *et al*. 2009 [11]	300	1	0	0.17%
3	Wattanasirichaigoon D, *et al*. 2004 [12]	166	6	2	3.01%
4	Padma G, *et al*. 2009 [13]	456	0	1	0.22%
5	Park HJ, *et al*. 2000 [14]	147	5	5	5.10%
6	Shalin H, *et al*. 2002 [15]	48	0	1	2.08%
7	Lee KY, *et al*. 2008 [16]	29	0	2	6.90%
8	Schimmenti LA, *et al*. 2008 [17]	95	0	1	1.05%
9	Posukh O, *et al*. 2005 [18]	76	4	1	3.95%
10	Abe S, *et al*. 2000 [19]	35	4	1	8.57%
11	Tang, HY *et al*. 2006 [20]	610	1	0	0.08%
12	Tekin M, *et al*. 2010 [21]	534	12	2	1.50%
13	Kudo T, *et al*. 2000 [22]	63	1	3	5.56%
15	Cheng X, *et al*. 2005 [23]	740	1	1	0.20%
16	Wang YC, et al. 2002 [24]	169	6	8	6.51%
17	Chen D, *et al*. 2009 [25]	100	10	16	21.00%
18	Xiao ZA, *et al*. 2004 [26]	131	2	9	7.63%
19	Wang SH, *et al*. 2009 [27]	140	7	20	16.79%
20	Hwa, HL *et al*. 2003 [28]	324	16	11	5.86%
21	Shi GZ, *et al*. 2004 [29]	20	3	3	22.50%
22	Dai P, *et al*. 2007 [30]	3004	255	233	12.00%
23	Liu ZX, *et al*. 2002 [31]	118	20	14	20.34%
24	Liu YH, *et al*.2002 [32]	210	13	27	15.95%

(*χ^2^ = *6.667, P <0.05.* A significant heterogeneity was found in the 235del mutation frequency of *GJB2* allele).

### Characteristics of included studies

A total of 36 case-control studies comprising 13217 patients’ cases with NSHL and 6521 controls were included in meta-analysis (Table 2). Each study was evaluated with a high score (not less than 5). Among them, there were 14 papers categorized to Grade A. All the included 36 case-control studies were consistent with the Hardy-Weinberg Equilibrium test, indicating a good representation of control populations.

**Table 2:** Relevant studies on 235delC mutation and NSHL

**No**	**Author**	**Score/ Grade**	**No**	**Author**	**Score/ Grade**
Studies in non-Asian populations					
1	Dahl HM, *et al*. 2006 [4]	6A	7	Cheng X, *et al*. 2005 [23]	5B
2	Ramsebner R, *et al*. 2007 [5]	5B	8	Utrera R, *et al*. 2007 [33]	5B
3	Batissoco AC, *et al*. 2009 [11]	5B	9	Tang HY, *et al*. 2006 [20]	6A
4	Damalon V, *et al*. 2010 [34]	5B	10	Pollak A, *et al*. 2007 [35]	5B
5	Samanich J, *et al*. 2007 [36]	5B	11	Tóth T, *et al*. 2007 [37]	5B
6	Schimmenti LA, *et al*. 2008 [17]	6A	12	Neocleous V, *et al*. 2006 [38]	5B
Studies in East Asian and Southeast Asian populations					
13	Snoeckx RL, *et al*. 2005 [39]	5B	23	Xiao ZA, *et al*. 2004 [26]	5B
14	Wattanasirichaigoon D, *et al*. 2004 [12]	5B	24	Wang SH, *et al*. 2009 [ 27]	6A
15	Park HJ, *et al*. 2000 [14]	6A	25	Hwa HL, *et al*. 2003 [28]	5B
16	Lee KY, *et al*. 2008 [16]	5B	26	Shi GZ, *et al*. 2004 [29]	6A
17	Abe S, *et al*. 2000 [19]	5B	27	Dai P, *et al*. 2007 [30]	6A
18	Ohtsuka A, *et al*. 2003 [10]	5B	28	Liu ZX, *et al*. 2002 [31]	5A
19	Tekin M , *et al*. 2010 [21]	5B	29	Liu YH, *et al*. 2002 [32]	6A
20	Kudo T, *et al*. 2000 [22]	5B	30	Chen GM, *et al*. 2011 [40]	5B
21	Wang YC, et al. 2002 [24]	6A	31	Guo YF, *et al*. 2008 [41]	6A
22	Chen D, *et al*. 2009 [25]	5B			
Studies in South Asian, West Asian and North Asian populations					
32	Padma G, *et al*. 2009 [13]	6A	35	Baysal E, *et al*. 2008 [42]	6A
33	Bhalla S, *et al*. 2009 [43]	6A	36	Posukh O, *et al*. 2005 [18]	5B
34	Shalin H, *et al*. 2002 [15]	5B			

### Meta-analysis of overall data

**Figure 2:**
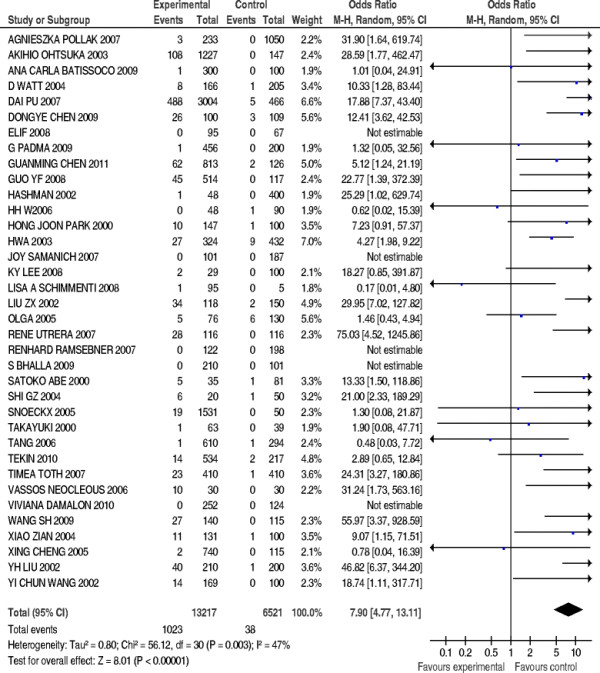
Forest plot on the association of 235delC mutation with the risk of NSHL.

**Figure 3:**
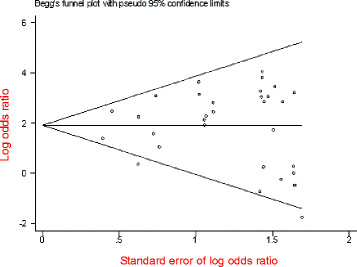
Begg’s funnel plot assessing publication bias on all included studies.

The 235delC mutation of *GJB2* gene was not detected in five case-control studies, so the rest of 31 studies compromising 12437 patients’ cases and 5844 controls were included in meta-anslysis. The pooled *OR* was calculated using random effect model, and the forest plot on the association of mutation carriers with the risk of NSHL was fulfilled (*OR* = 7.9, 95%*CI*: 4.77~13.11, *Z* = 8.01, *P*<0.00001). As shown in Figure 2, the pooled *OR* and 95%*CI* values demonstrated that there was a higher prevalence of the 235delC mutation in the case group than in the control group. The publication bias was examined by use of funnel plot (Figure 3). The visual inspection of funnel plot seemed to be symmetrical, and most of the data points were located within 95%*CI*. However, an absence of corner was observed possibly due to the unpublished negative results. Additionally, the Begg’s test was conducted to analyze the symmetry of the funnel plot, and no significant publication bias was found (*N*_fs_ = 58.84>10, *P* = 0.341>0.05), which suggested that the publication bias had little effect on the result of meta-analysis and the conclusions obtained were confident.

**Figure 4:**
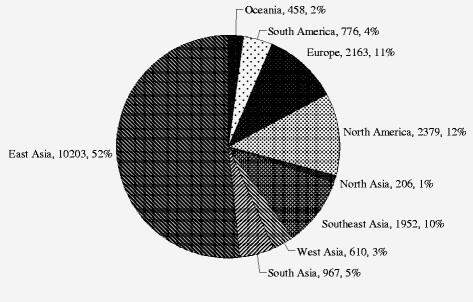
Proportions of tested individuals in different continents.

Heterogeneity of genetic effect was detected as shown in Figure 2 (χ^2^ = 56.12, *df* = 30, *P =* 0.003<0.05, *I*^*2*^ = 47%), which indicated that the association of the 235delC mutation with the risk of NSHL was varied due to the ethnic specificity or regional disparity. To elucidate the prevalence of the 235delC mutation related to the NSHL susceptibility more precisely, the stratified meta-analysis was subsequently performed according to the regional studies. It was observed that a high prevalence of the 235delC mutation related to hearing loss was reported in Asian (Figure 4). Therefore, the populations of the included studies were divided into Asian and Non-Asian population groups, and the stratified meta-analysis was conducted to each group.

### Stratified meta-analysis of Asian population group

**Figure 5:**
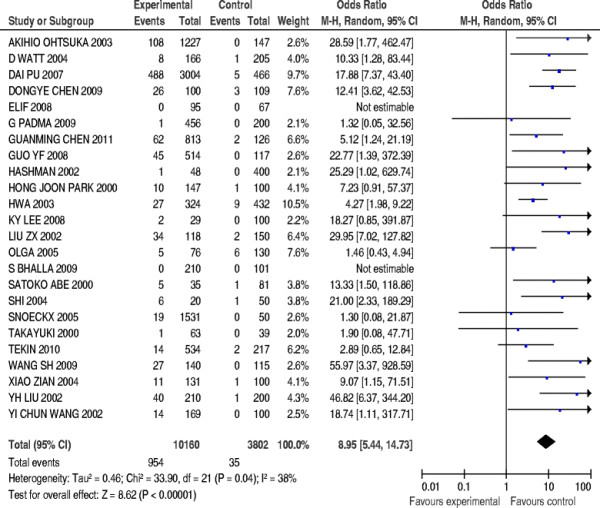
Forest plot on the association of 235delC mutation with the risk of NSHL in Asian populations.

**Figure 6:**
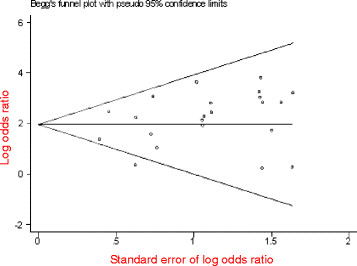
Begg’s funnel plot assessing publication bias on included studies in Asian populations.

Two studies were excluded due to undetectable 235delC mutations in patients’ cases or incomplete statistical data in controls. And a total of 22 papers compromising 9855 patients’ cases and 3634 controls were included in the stratified meta-analysis. The pooled *OR* was calculated using random effect model, and forest plot on the association of mutation carriers with the risk of NSHL was fulfilled (*OR* = 8.95, 95%*CI*: 5.44 ~ 14.73, *Z* = 8.62, *P* < 0.00001). As shown in Figure 5, the pooled *OR* and 95%*CI* values demonstrated that the 235delC mutation of *GJB2* gene was significantly associated with the risk of NSHL in Asian population. The visual inspection of funnel plot seemed to be symmetrical (Figure 6), and most of the data points were located within 95%*CI*. The Begg’s test was also used to analyze the symmetry of funnel plot, and no significant publication bias was found (*N*_fs_ = 35.46>10, *P* = 0.499>0.05), which suggested that and the publication bias had little effect on the result of meta-analysis and the conclusions obtained were confident.

Heterogeneity of genetic effect was still detected as shown in Figure 5 (χ^2^ = 33.90, *df* = 21, *P =* 0.04<0.05, *I*^*2*^ = 38%), which demonstrated the presence of disparity in the prevalence of the 235delC mutation related to NSHL susceptibility among Asian populations suggesting the need of further stratified meta-analysis.

### Stratified Meta-analysis of East Asian and Southeast Asian populations

**Figure 7:**
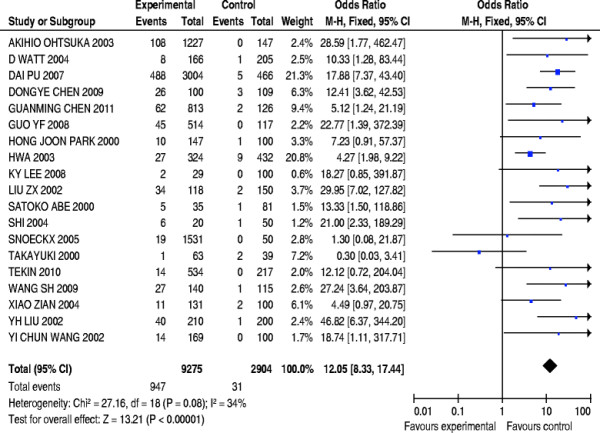
Forest plot on the association of 235delC mutation with the risk of NSHL in East Asian and Southeast Asian populations.

**Figure 8:**
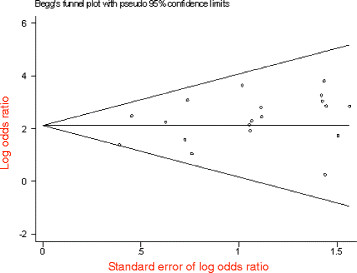
Begg’s funnel plot assessing publication bias on included studies in East Asian and Southeast Asian populations.

The overall effect on included studies in East Asian and Southeast Asian populations was evaluated, and a total of 19 papers were included, compromising 9275 patients’ cases and 2904 controls. The forest plot on the association of mutation carriers with the risk of NSHL was fulfilled using random effect model (*OR* = 12.05, 95%*CI*: 8.33 ~ 17.44, *Z* = 13.21, *P* <0.00001). As shown in Figure 7, the pooled *OR* and 95%*CI* values demonstrated that the prevalence of the 235delC mutation was higher in the case group than in the control group. Publication bias was examined by use of funnel plot (Figure 8). The Begg’s test was also used to analyze the symmetry of funnel plot, and no significant publication bias was found (*N*_fs_ = 28.58>10, *P* = 0.576>0.05), which suggested that and the publication bias had little effect on the result of meta-analysis and the obtained conclusion was confident. In addition, no significant heterogeneity of genetic effect was found as shown in Figure 7 (χ^2^ = 27.16, *df* = 18, *P =* 0.08>0.05, *I*^*2*^ = 34%).

The above analytic results indicated that the 235delC mutation of *GJB2* gene was significantly associated with the risk of NSHL in East Asian and Southeast Asian. Due to the insufficient searched studies, the meta-analysis was not performed to the regional studies in North Asian, West Asian and South Asian populations, respectively.

### Stratified Meta-analysis of Non-Asian population group (Europe and Oceania populations)

**Figure 9:**
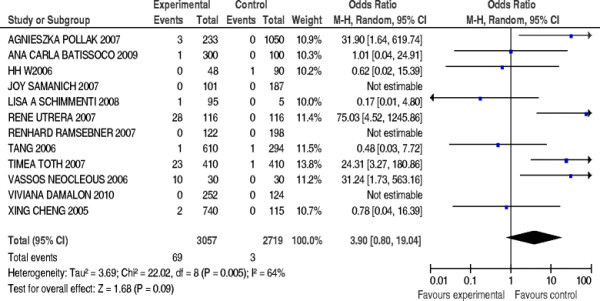
Forest plot on the association of 235delC mutation with the risk of NSHL in Europe and Oceania populations.

Three case-control studies were excluded due to no 235delC mutation of *GJB2* gene detected, and a total of 9 papers were included, compromising 2683 patients’ cases and 2397 controls. The pooled *OR* was calculated using random effect model, and forest plot on the association of mutation carriers with the risk of NSHL was fulfilled (Figure 9). The prevalence of the 235delC mutation seemed to be ethnic-specific, but was not significantly associated with the NSHL susceptibility in European and Oceania population (*OR* = 10.36, 95%*CI*: 4.68 ~ 22.96, *Z* = 1.68, *P* = 0.09>0.05).

## Discussion

The investigation on heterogeneity in genetic association were commonly leveled to the genetic effect and genetic model: the genetic effect focused on the difference in study method, potential interaction of gene-gene or gene-environment and linkage disequilibrium [13], while the genetic model was mainly concerned with the differential association between heterozygote (or homozygote) and the risk of human diseases. The development of hearing loss involved with numerous factors and experienced multiple steps, and the interaction of heredity (genes) and environmental factors played an important role, which could have a potential influence on the association of *GJB2* mutations and the NSHL susceptibility with ethnic specificity or certain genetic backgrounds of affected families. Meanwhile, the same type of single nucleotide polymorphisms (SNP) could show various contributions to the genetic models derived from different studies due to the ethnic specificity.

By increasing the sample size, meta-analysis based on case-control studies has the potential to evaluate the between-study heterogeneity and detect small effects in genetic association. In this study, the combined results of meta-analysis showed that the 235delC mutant increased the risk of NSHL, but significant between-study heterogeneity and genetic-effect heterogeneity were detected, indicating that the ethnic specificity and regional disparity contributed to the association between the 235delC mutation of *GJB2* gene and the NSHL susceptibility. Results of stratified meta-analysis showed that the 235delC mutation was prominently associated with the risk of NSHL in East Asian and Southeast Asian populations, but not significantly in European and Oceania populations. The 235delC mutation of *GJB2* gene trended to be consistent with recessive model in Chinese population, while heterogeneity of genetic effect was also detected in studies of *GJB2* mutation reported in Europe and Oceania, which suggested that multiple models could possibly be present in European and American populations, due to a complex interaction between *GJB2* mutations and environmental factors. A further research on the prevalence of the 235delC mutation of *GJB2* gene related to the NSHL susceptibility, as well as the regional disparity and ethnic specificity will be essentially need.

## Conclusions

A systematic review was performed by means of a meta-analysis to evaluate the influence of the 235delC mutation on the NSHL susceptibility. The combined results of meta-analysis by pooling all relevant studies showed that the 235delC mutant increased the risk of NSHL. However, heterogeneity of genetic effect was observed due to the ethnic specificity and regional disparity. Therefore, stratified meta-analysis was conducted in different ethnic population group, and the results indicated that the 235delC mutation was prominently correlated with the risk of NHSL in the East Asian and South-east Asian populations, but non-significantly associated with the Oceania and European populations. Based on this study, a further research on the genetic mechanism of the NSHL susceptibility related to the 235delC mutation of *GJB2* gene will be carried out in our future work.

## Competing interests

The authors declare that they have no competing interests.

## Authors’ contributions

JY and YJL carried out the literature search and screening, performed the meta-analysis and drafted the manuscript. QJW participated in the design of the study and the statistical analysis. GQX and XC conceived of the design of the study and helped to draft the manuscript. All authors read and approved the final manuscript.
